# 2-Hy­droxy-*N*-(4-methyl­phen­yl)benzamide

**DOI:** 10.1107/S1600536811030716

**Published:** 2011-08-06

**Authors:** Abdul Rauf Raza, Bushra Nisar, M. Nawaz Tahir

**Affiliations:** a Department of Chemistry, University of Sargodha, Sargodha, Pakistan; b Department of Physics, University of Sargodha, Sargodha, Pakistan

## Abstract

In the crystal structure of the title compound, C_14_H_13_NO_2_, the mol­ecules are approximately planar, the r.m.s. deviation for all non-H atoms being 0.0435 Å; the dihedral angle between the two rings is 3.45 (12)°. The planarity is accounted for in terms of the presence of intra­molecular N—H⋯O and C—H⋯O hydrogen bonding, each of which completes an *S*(6) ring motif. The mol­ecules are stabilized in the form of supra­molecular chains extending along the crystallographic *c* axis due to inter­molecular O—H⋯O and C—H⋯O hydrogen bonding; each type leads to an *R*_2_^1^(6) ring motif.

## Related literature

For related benzamide structures, see: Raza *et al.* (2010*a* ▶,*b* ▶,*c* ▶). For graph-set notation, see: Bernstein *et al.* (1995 ▶).
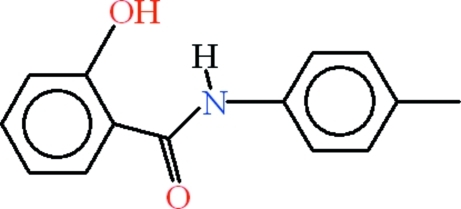


## Experimental

### 

#### Crystal data

C_14_H_13_NO_2_
*M* *_r_* = 227.25
Monoclinic, 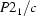

*a* = 19.4067 (17) Å
*b* = 4.9122 (5) Å
*c* = 12.7261 (11) Å
β = 104.793 (4)°
*V* = 1172.96 (19) Å^3^
*Z* = 4
Mo *K*α radiation
μ = 0.09 mm^−1^
*T* = 296 K
0.34 × 0.14 × 0.12 mm

#### Data collection

Bruker Kappa APEXII CCD diffractometer
Absorption correction: multi-scan (*SADABS*; Bruker, 2009 ▶) *T*_min_ = 0.979, *T*_max_ = 0.988
10416 measured reflections
2771 independent reflections
1243 reflections with *I* > 2σ(*I*)
*R*_int_ = 0.060

#### Refinement

*R*[*F* ^2^ > 2σ(*F* ^2^)] = 0.059
*wR*(*F* ^2^) = 0.159
*S* = 0.96
2771 reflections
156 parameters
H-atom parameters constrained
Δρ_max_ = 0.19 e Å^−3^
Δρ_min_ = −0.16 e Å^−3^

### 

Data collection: *APEX2* (Bruker, 2009 ▶); cell refinement: *SAINT* (Bruker, 2009 ▶); data reduction: *SAINT*; program(s) used to solve structure: *SHELXS97* (Sheldrick, 2008 ▶); program(s) used to refine structure: *SHELXL97* (Sheldrick, 2008 ▶); molecular graphics: *ORTEP-3 for Windows* (Farrugia, 1997 ▶) and *PLATON* (Spek, 2009 ▶); software used to prepare material for publication: *WinGX* (Farrugia, 1999 ▶) and *PLATON*.

## Supplementary Material

Crystal structure: contains datablock(s) global, I. DOI: 10.1107/S1600536811030716/tk2771sup1.cif

Structure factors: contains datablock(s) I. DOI: 10.1107/S1600536811030716/tk2771Isup2.hkl

Supplementary material file. DOI: 10.1107/S1600536811030716/tk2771Isup3.cml

Additional supplementary materials:  crystallographic information; 3D view; checkCIF report

## Figures and Tables

**Table 1:** Hydrogen-bond geometry (Å, °)

*D*—H⋯*A*	*D*—H	H⋯*A*	*D*⋯*A*	*D*—H⋯*A*
O1—H1⋯O2^i^	0.82	1.78	2.596 (2)	179
N1—H1*A*⋯O1	0.86	1.92	2.647 (2)	141
C3—H3⋯O2^i^	0.93	2.51	3.179 (3)	129
C9—H9⋯O2	0.93	2.25	2.840 (3)	121

Symmetry code: (i) 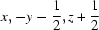.

**Fig. 1.**
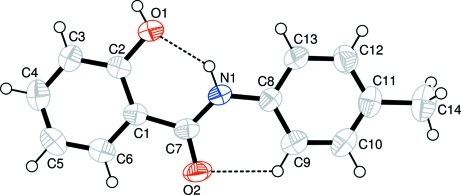
View of the title compound with the atom numbering scheme. The displacement ellipsoids are drawn at the 50% probability level. H-atoms are shown by small circles of arbitrary radii. The dotted line indicate the intramolecular H-bonding.

**Fig. 2.**
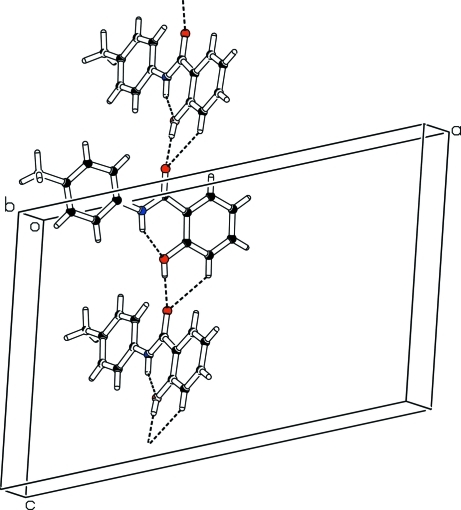
The partial packing diagram which shows that molecules form supramolecular chains extending along the c-axis. The dotted line indicate the intramolecular H-bonding.

## supplementary crystallographic information

### Comment

We have reported the crystal structures of (II) *i.e*. 2-hydroxy-*N*-(3-nitrophenyl)benzamide (Raza *et al.*, 2010*a*), (III) *i.e*. *N*-(4-chlorophenyl)-2-hydroxybenzamide (Raza *et al.*, 2010*b*) and (IV) *i.e*. *N*-(3-chlorophenyl)-2 -hydroxybenzamide (Raza *et al.*, 2010*c*). In this connection, the title compound (I, Fig. 1) has been prepared as a precursor for the synthesis of symmetric as well as asymmetric benzoxazepines.

In (I), the 2-hydroxyphenyl group A (C1–C6/O1) and 4-methylanilinic group B (C8—C14/N1) are planar with r.m.s. deviations of 0.0048 and 0.0086 Å, respectively. The dihedral angle between A/B is 3.45 (12) °. There is intramolecular H-bonding of the type N—H···O and C—H···O types (Table 1, Fig. 1), each of which completes a *S*(6) ring motif (Bernstein *et al.*, 1995). There is also intermolecular H-bonding of the type C—H···O and O—H···O (Table 1). These lead to the formation of two *R*_2_^1^(6) ring motifs and to supramolecular chains extending along the crystallographic *c*-axis (Fig. 2).

### Experimental

To a well stirred solution of 2-hydroxy benzoic acid (2.76 g, 0.02 mol, 1 equiv.) and SOCl_2_ (1.74 mL, 2.84 g, 0.024 mol, 1.2 equiv.) in dry CHCl_3_, 4-metylaniline (2.14 g, 0.02 mol, 1 equiv.) and Et_3_N (4.16 mL, 3 g, 0.03 mol, 1.5 equiv.) were added slowly at room temperature followed by 3 h reflux. After completion of reaction, the reaction mixture was cooled to room temperature, neutralized with aqueous NaHCO_3_ (10 %) and extracted with CHCl_3_ (3×25 mL). The organic layers were combined, dried over anhydrous Na_2_SO_4_, filtered and concentrated under reduced pressure to afford a brown solid. The column chromatographic purification with 1%, 2% and 3% CHCl_3_ in petrol (300 mL each) over a silica gel packed column (of 25.5 cm length) afforded white needles of (I) in the 96th-280th fractions (50 mL each).

### Refinement

Although H atoms appeared in difference Fourier maps they were positioned geometrically with (O–H = 0.82, N–H = 0.86 and C–H = 0.93-0.96 Å) and refined as riding with *U*_iso_(H) = x*U*_eq_(C), where x = 1.5 for hydroxy- & methyl-H atoms and x = 1.2 for other H atoms.

### Crystal data

**Table:**

C_14_H_13_NO_2_	*F*(000) = 480
*M**_r_* = 227.25	*D*_x_ = 1.287 Mg m^−^^3^
Monoclinic, *P*2_1_/*c*	Mo *K*α radiation, λ = 0.71073 Å
Hall symbol: -P 2ybc	Cell parameters from 1243 reflections
*a* = 19.4067 (17) Å	θ = 1.1–27.9°
*b* = 4.9122 (5) Å	µ = 0.09 mm^−^^1^
*c* = 12.7261 (11) Å	*T* = 296 K
β = 104.793 (4)°	Needle, colorless
*V* = 1172.96 (19) Å^3^	0.34 × 0.14 × 0.12 mm
*Z* = 4	

### Data collection

**Table:**

Bruker Kappa APEXII CCD diffractometer	2771 independent reflections
Radiation source: fine-focus sealed tube	1243 reflections with *I* > 2σ(*I*)
graphite	*R*_int_ = 0.060
Detector resolution: 7.6 pixels mm^-1^	θ_max_ = 27.9°, θ_min_ = 1.1°
ω scans	*h* = −25→25
Absorption correction: multi-scan (*SADABS*; Bruker, 2009)	*k* = −4→6
*T*_min_ = 0.979, *T*_max_ = 0.988	*l* = −16→16
10416 measured reflections	

### Refinement

**Table:**

Refinement on *F*^2^	Primary atom site location: structure-invariant direct methods
Least-squares matrix: full	Secondary atom site location: difference Fourier map
*R*[*F*^2^ > 2σ(*F*^2^)] = 0.059	Hydrogen site location: inferred from neighbouring sites
*wR*(*F*^2^) = 0.159	H-atom parameters constrained
*S* = 0.96	*w* = 1/[σ^2^(*F*_o_^2^) + (0.0648*P*)^2^] where *P* = (*F*_o_^2^ + 2*F*_c_^2^)/3
2771 reflections	(Δ/σ)_max_ < 0.001
156 parameters	Δρ_max_ = 0.19 e Å^−^^3^
0 restraints	Δρ_min_ = −0.16 e Å^−^^3^

### Special details

**Table:**

Geometry. Bond distances, angles etc. have been calculated using the rounded fractional coordinates. All su's are estimated from the variances of the (full) variance-covariance matrix. The cell esds are taken into account in the estimation of distances, angles and torsion angles
Refinement. Refinement of *F*^2^ against ALL reflections. The weighted *R*-factor *wR* and goodness of fit *S* are based on *F*^2^, conventional *R*-factors *R* are based on *F*, with *F* set to zero for negative *F*^2^. The threshold expression of *F*^2^ > σ(*F*^2^) is used only for calculating *R*-factors(gt) *etc*. and is not relevant to the choice of reflections for refinement. *R*-factors based on *F*^2^ are statistically about twice as large as those based on *F*, and *R*- factors based on ALL data will be even larger.

### Fractional atomic coordinates and isotropic or equivalent isotropic displacement parameters (Å^2^)

**Table:**

	*x*	*y*	*z*	*U*_iso_*/*U*_eq_	
O1	0.29367 (9)	−0.2157 (4)	0.22720 (13)	0.0599 (7)	
O2	0.29429 (9)	−0.0348 (3)	−0.09273 (12)	0.0598 (7)	
N1	0.24804 (9)	0.0722 (4)	0.04697 (14)	0.0438 (7)	
C1	0.33925 (11)	−0.2782 (5)	0.07185 (18)	0.0407 (8)	
C2	0.33876 (12)	−0.3489 (5)	0.17802 (18)	0.0438 (8)	
C3	0.38382 (13)	−0.5515 (5)	0.2327 (2)	0.0516 (9)	
C4	0.42941 (13)	−0.6849 (5)	0.1833 (2)	0.0596 (11)	
C5	0.43143 (13)	−0.6168 (6)	0.0796 (2)	0.0615 (11)	
C6	0.38654 (13)	−0.4161 (5)	0.0249 (2)	0.0539 (10)	
C7	0.29213 (12)	−0.0712 (5)	0.00262 (18)	0.0416 (8)	
C8	0.19724 (12)	0.2725 (5)	−0.00178 (18)	0.0428 (8)	
C9	0.18909 (14)	0.3743 (5)	−0.1055 (2)	0.0564 (10)	
C10	0.13704 (14)	0.5697 (6)	−0.1449 (2)	0.0605 (11)	
C11	0.09251 (13)	0.6670 (5)	−0.0859 (2)	0.0544 (10)	
C12	0.10233 (14)	0.5654 (5)	0.0186 (2)	0.0592 (10)	
C13	0.15368 (13)	0.3727 (5)	0.06023 (19)	0.0524 (9)	
C14	0.03636 (15)	0.8783 (5)	−0.1313 (2)	0.0751 (11)	
H1	0.29314	−0.29364	0.28396	0.0899*	
H1A	0.25091	0.03812	0.11424	0.0525*	
H3	0.38315	−0.59736	0.30329	0.0619*	
H4	0.45896	−0.82174	0.22036	0.0714*	
H5	0.46273	−0.70494	0.04648	0.0739*	
H6	0.38794	−0.37165	−0.04556	0.0647*	
H9	0.21810	0.31275	−0.14848	0.0677*	
H10	0.13229	0.63728	−0.21461	0.0726*	
H12	0.07355	0.62883	0.06158	0.0711*	
H13	0.15915	0.30919	0.13074	0.0628*	
H14A	0.03008	0.89281	−0.20839	0.1127*	
H14B	0.05114	1.05104	−0.09782	0.1127*	
H14C	−0.00787	0.82537	−0.11655	0.1127*	

### Atomic displacement parameters (Å^2^)

**Table:**

	*U*^11^	*U*^22^	*U*^33^	*U*^12^	*U*^13^	*U*^23^
O1	0.0753 (12)	0.0677 (13)	0.0429 (11)	0.0164 (10)	0.0262 (9)	0.0130 (9)
O2	0.0889 (13)	0.0586 (13)	0.0371 (10)	0.0035 (10)	0.0256 (9)	0.0009 (8)
N1	0.0496 (12)	0.0504 (14)	0.0318 (10)	0.0029 (10)	0.0111 (9)	0.0039 (9)
C1	0.0437 (13)	0.0379 (15)	0.0409 (14)	−0.0064 (11)	0.0117 (11)	−0.0060 (11)
C2	0.0449 (14)	0.0444 (16)	0.0438 (14)	−0.0034 (12)	0.0147 (12)	−0.0036 (12)
C3	0.0500 (14)	0.0479 (18)	0.0534 (16)	−0.0010 (13)	0.0070 (12)	0.0053 (13)
C4	0.0506 (16)	0.0481 (19)	0.077 (2)	0.0041 (13)	0.0109 (15)	0.0031 (14)
C5	0.0525 (16)	0.061 (2)	0.076 (2)	0.0033 (15)	0.0257 (15)	−0.0090 (16)
C6	0.0557 (16)	0.0571 (19)	0.0522 (16)	−0.0014 (14)	0.0197 (13)	−0.0048 (14)
C7	0.0494 (14)	0.0400 (16)	0.0365 (14)	−0.0101 (12)	0.0132 (11)	−0.0050 (11)
C8	0.0474 (14)	0.0383 (15)	0.0400 (14)	−0.0038 (12)	0.0062 (11)	0.0006 (11)
C9	0.0608 (16)	0.0616 (19)	0.0465 (16)	−0.0014 (15)	0.0130 (13)	0.0051 (14)
C10	0.0673 (18)	0.056 (2)	0.0515 (17)	−0.0024 (15)	0.0028 (14)	0.0110 (14)
C11	0.0544 (16)	0.0360 (16)	0.0628 (19)	−0.0060 (13)	−0.0031 (14)	−0.0021 (13)
C12	0.0613 (17)	0.0524 (19)	0.0615 (18)	0.0060 (14)	0.0112 (14)	−0.0038 (14)
C13	0.0599 (16)	0.0563 (18)	0.0397 (14)	0.0043 (14)	0.0105 (12)	0.0046 (12)
C14	0.0718 (19)	0.0511 (19)	0.086 (2)	0.0023 (16)	−0.0097 (16)	0.0010 (16)

### Geometric parameters (Å, °)

**Table:**

O1—C2	1.366 (3)	C10—C11	1.368 (4)
O2—C7	1.238 (3)	C11—C12	1.387 (3)
O1—H1	0.8200	C11—C14	1.509 (4)
N1—C7	1.339 (3)	C12—C13	1.379 (4)
N1—C8	1.419 (3)	C3—H3	0.9300
N1—H1A	0.8600	C4—H4	0.9300
C1—C7	1.495 (3)	C5—H5	0.9300
C1—C6	1.392 (3)	C6—H6	0.9300
C1—C2	1.397 (3)	C9—H9	0.9300
C2—C3	1.389 (3)	C10—H10	0.9300
C3—C4	1.375 (4)	C12—H12	0.9300
C4—C5	1.372 (4)	C13—H13	0.9300
C5—C6	1.381 (4)	C14—H14A	0.9600
C8—C13	1.386 (3)	C14—H14B	0.9600
C8—C9	1.382 (3)	C14—H14C	0.9600
C9—C10	1.391 (4)		
			
C2—O1—H1	109.00	C11—C12—C13	121.6 (2)
C7—N1—C8	128.90 (19)	C8—C13—C12	120.8 (2)
C8—N1—H1A	116.00	C2—C3—H3	120.00
C7—N1—H1A	116.00	C4—C3—H3	120.00
C2—C1—C6	117.5 (2)	C3—C4—H4	120.00
C2—C1—C7	125.8 (2)	C5—C4—H4	120.00
C6—C1—C7	116.7 (2)	C4—C5—H5	120.00
O1—C2—C3	120.6 (2)	C6—C5—H5	120.00
O1—C2—C1	119.2 (2)	C1—C6—H6	119.00
C1—C2—C3	120.3 (2)	C5—C6—H6	119.00
C2—C3—C4	120.6 (2)	C8—C9—H9	120.00
C3—C4—C5	120.2 (2)	C10—C9—H9	120.00
C4—C5—C6	119.3 (2)	C9—C10—H10	118.00
C1—C6—C5	122.1 (2)	C11—C10—H10	118.00
N1—C7—C1	118.03 (19)	C11—C12—H12	119.00
O2—C7—C1	120.5 (2)	C13—C12—H12	119.00
O2—C7—N1	121.5 (2)	C8—C13—H13	120.00
N1—C8—C9	124.5 (2)	C12—C13—H13	120.00
N1—C8—C13	117.0 (2)	C11—C14—H14A	109.00
C9—C8—C13	118.5 (2)	C11—C14—H14B	109.00
C8—C9—C10	119.3 (2)	C11—C14—H14C	109.00
C9—C10—C11	123.1 (2)	H14A—C14—H14B	109.00
C10—C11—C14	121.7 (2)	H14A—C14—H14C	109.00
C10—C11—C12	116.7 (2)	H14B—C14—H14C	109.00
C12—C11—C14	121.6 (2)		
			
C8—N1—C7—O2	2.0 (4)	C1—C2—C3—C4	0.0 (4)
C8—N1—C7—C1	−178.1 (2)	C2—C3—C4—C5	0.8 (4)
C7—N1—C8—C9	−6.3 (4)	C3—C4—C5—C6	−1.0 (4)
C7—N1—C8—C13	174.3 (2)	C4—C5—C6—C1	0.4 (4)
C6—C1—C2—O1	179.2 (2)	N1—C8—C9—C10	179.6 (2)
C6—C1—C2—C3	−0.5 (4)	C13—C8—C9—C10	−1.0 (4)
C7—C1—C2—O1	−2.3 (4)	N1—C8—C13—C12	−179.2 (2)
C7—C1—C2—C3	177.9 (2)	C9—C8—C13—C12	1.4 (4)
C2—C1—C6—C5	0.3 (4)	C8—C9—C10—C11	−0.4 (4)
C7—C1—C6—C5	−178.3 (2)	C9—C10—C11—C12	1.4 (4)
C2—C1—C7—O2	−176.0 (2)	C9—C10—C11—C14	−179.7 (2)
C2—C1—C7—N1	4.1 (4)	C10—C11—C12—C13	−1.0 (4)
C6—C1—C7—O2	2.5 (3)	C14—C11—C12—C13	−179.9 (2)
C6—C1—C7—N1	−177.5 (2)	C11—C12—C13—C8	−0.4 (4)
O1—C2—C3—C4	−179.8 (2)		

### Hydrogen-bond geometry (Å, °)

**Table:**

*D*—H···*A*	*D*—H	H···*A*	*D*···*A*	*D*—H···*A*
O1—H1···O2^i^	0.82	1.78	2.596 (2)	179
N1—H1A···O1	0.86	1.92	2.647 (2)	141
C3—H3···O2^i^	0.93	2.51	3.179 (3)	129
C9—H9···O2	0.93	2.25	2.840 (3)	121

Symmetry codes: (i) *x*, −*y*−1/2, *z*+1/2.
